# Assessing the Impact of a Game-Centered Mobile App on Community-Dwelling Older Adults′ Health Activation

**DOI:** 10.21926/obm.icm.1903041

**Published:** 2019-07-04

**Authors:** K. Jason Crandall, Matthew Shake, Uta Ziegler

**Affiliations:** 1. Western Kentucky University Center for Applied Science in Health and Aging, 2413 Nashville Road Suite, 123, Bowling Green, USA;; 2. Western Kentucky University Department of Psychological Sciences, 1906 College Heights Blvd, KTH 1002, Bowling Green, USA;; 3. Western Kentucky University School of Engineering and Applied Science, 1906 College Heights Blvd., COHH 4036, Bowling Green, USA;

**Keywords:** Health activation, health promotion, exercise, older adults, health education, technology, falls risk reduction, osteoarthritis

## Abstract

**Background:**

Older adults experience normative age-graded declines in physical and cognitive performance and many must manage one or more chronic conditions. Exercise programs can help to improve both their physical health and their knowledge, skill, and confidence in managing aspects of their own healthcare, yet a significant barrier is motivating them to adhere to such programs. The purpose of this investigation was to evaluate the impact of a game-centered mobile app (Bingocize®) on older adults’ knowledge, skill, and confidence for managing aspects of their healthcare.

**Methods:**

Community-dwelling older adults (N=84) with mobility and not engaged in any structured exercise program were recruited from rural community senior centers in Kentucky and Tennessee. Participants were randomly assigned to (a) a version that included health education, or (b) health education and an exercise component. Participants used the app in a group setting for 10 weeks, twice per week, for one hour. The Patient Activation Measure (PAM-10) was used to assess group changes in knowledge, skill, and confidence for managing aspects of their healthcare. The design was a two (Group: Exercise + Health Education vs. Health Education-only) x two (Time: Pre- vs. Post-intervention) and an analyses of variance, with significance p<.05, was used to detect within and between group differences.

**Results:**

PAM-10 values significantly increased from pre- to post-intervention for both groups, as did knowledge of the health topics (all p < 0.05). Attendance was >93% in both groups.

**Conclusions:**

Bingocize® engendered high attendance and improved health activation of older adults; however, additional research is needed to examine whether changes in activation result in long-term changes in health behaviour. The Bingocize® mobile app is an enjoyable and effective way to increase health activation in community-dwelling older adults.

## 1. Introduction

Older adults’ health-related challenges are significant. In addition to normative age-graded declines in aspects of physical and cognitive performance [1], they often must manage one or more chronic conditions [2]. There is convincing evidence that health promotion programs (e.g., exercise programs geared toward older adults) can help older adults better manage their chronic conditions by improving health activation. Health activation is defined as the knowledge, skill, and confidence in managing aspects of their own healthcare [3]. However, a significant barrier is motivating them to adhere to such programs [4]. Adherence is often low in health promotion programs for several reasons; for example, older adults often perceive such programs as negative and/or painful experiences, and many existing programs are solitary in nature despite the fact that research suggests older adults are more motivated to change health behaviours in group or social settings [4]. It is well-known that the level of active engagement a patient takes in their own healthcare is a critical factor in the success of any health intervention. As such, increasing the self-management abilities of older adults has the potential to improve quality of life (including overall well-being, better health-care outcomes and care experiences [5], and reduce financial stressors on the health-care system [6]. Yet, the overall amount of healthcare activation that older adults possess varies greatly between individuals [7]. One commonly used, broad measure to assess and quantify a person’s combination of knowledge, skills, and confidence to manage one’s healthcare is the Patient Activation Measure (PAM-10) [8], which has been applied to patients and non-patients alike [9–11]. The PAM-10 segments individuals into one of four levels, with a low PAM-10 score (1 out of 4) indicating that a person is passive and lacks knowledge and confidence to take action on their health, while the highest score (level 4) indicates a person is adopting health-promoting behaviours and actively advocates for themselves in healthcare decisions [12]. An example of one of the 10 statements is, “I know what each of my medications do.” Researchers have shown that health promotion interventions, such as Stanford’s Chronic Disease Self-Management Program (CDSMP), can lead to increases in PAM-10 scores [2, 6, 13, 14] and that increases in PAM-10 scores predict desirable modifications of healthy behaviour, better health outcomes, and reduced health-care costs (6, 14). PAM-10 scores are a valid assessment of health activation across different diseases, conditions, patients’ economic backgrounds, and nationalities [5, 15]. Despite the fact there are existing beneficial interventions for older adults, many fail to attract and retain the most vulnerable older adults, specifically, those in poor health [16,17].

Bingo is a popular game among many older adults, making it an ideal way to motivate them to participate in health promoting activities. With this information in mind, a mobile app (Bingocize®) played on tablets/mobile phones was created. Bingocize® socially engages users in a bingo-like game that can be modified to deliver exercise or health education (unimodal) or a combination of the two (multimodal). Bingocize® was shown in recent studies to lead to increased knowledge of health topics (osteoarthritis, falls risk reduction) and to engender a variety of positive health benefits among populations of sedentary, community-dwelling older adults [18]. Osteoarthritis was a focus of the health education component of the previous investigation because it is the leading cause of chronic disability in the developed world [19]. Falls risk reduction was also a focus because in 2012 there were over 3.2 million medically treated non-fatal falls in the United States [20].

The present study evaluated the impact of Bingocize® on health activation of rural community-dwelling older adults. Specifically, we hypothesized a multimodal version of Bingocize® that combines exercise and health education would show greater increases in health activation compared to a unimodal approach that uses health education alone.

## 2. Materials and Methods

**Figure 1:**
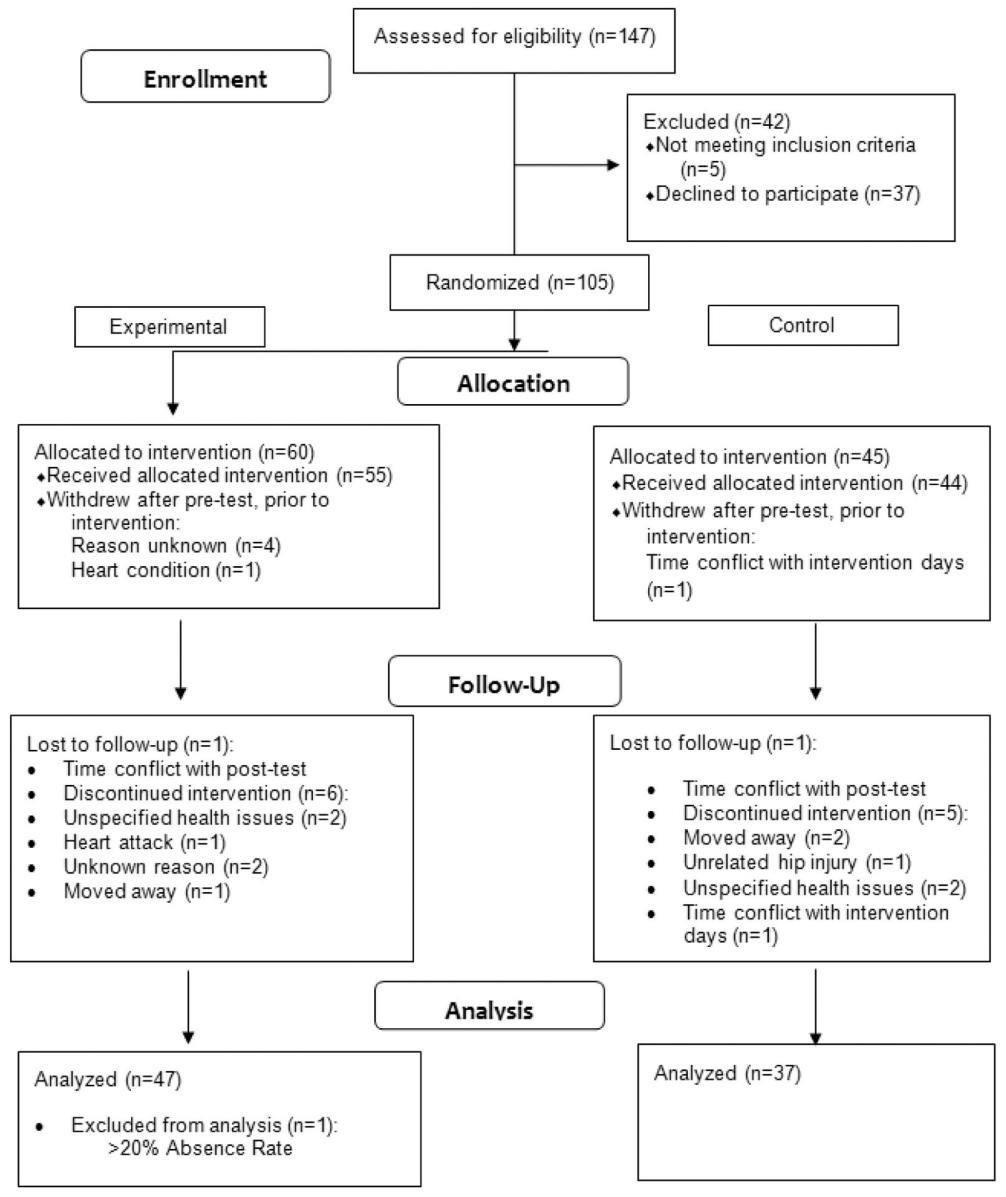
CONSORT diagram flow of participants through investigation.

Using direct contact, flyers, and word of mouth, we recruited 147 participants from 10 community senior centers located in Kentucky and Tennessee. A CONSORT diagram (Figure 1) proves the recruitment and assignment process of the current study. Of the 147 recruited, 105 (71%) met study requirements and gave informed consent. Of those, 84 (80%) completed the study. Study requirements for inclusion were the following: a minimum score of 17 on an adapted version of the telephone mini-mental status exam (T-MMSE), normal or corrected-normal vision, no severe neurological impairment, mobility (i.e., not wheelchair-bound), no colorblindness, had not been participating in any regular physical activity program for the prior six months, and English as a native language [21]. Participants were compensated $40. See Table 1 for baseline demographics.

### 2.1 Design

Prior to study initiation, approval was obtained from the Western Kentucky University’s (WKU) Institutional Review Board, and all participants were treated in accordance with the ethical principles and code of conduct of the American Psychological Association [22]. The study used a 2 (Group: Exercise + Health Education) vs. Health Education only) by 2 (Time: Pre- vs. Post-Intervention) design with senior center groups being randomly assigned to condition immediately after pre-intervention measures were collected. Random assignment of participants to condition was not practical; instead, groups of participants from each of the 10 senior centers were randomly assigned by a colleague not involved in the investigation, using a table of random numbers, to one of the two conditions. Due to small differences in group (cluster) sizes, random assignment resulted in n=60 being allocated to the Experimental Arm and n=45 being allocated to the Control Arm. Pre- and post-intervention measures were collected individually, with each participant coming to their community senior center at an appointed time. The pre- and post-intervention measures were administered and collected by trained graduate and undergraduate students who were blind to condition assignment.

### 2.2 Measures

To assess health activation, the PAM-10 questionnaire was administered to each participant within one week of the start of the program and again within one week after the end of the 10-week program. The PAM-10 includes 10 items which are scored (0–100 scale) and then used to categorize an individual on a continuum ranging from level 1 (lowest) to level 4 (highest) in terms of health activation. Additional physical and cognitive performance tests were also administered; details about those tests can be found in Shake et al. [18].

To assess participants’ knowledge of the two health topics (osteoarthritis and falls risk reduction), a 30-question health knowledge test was administered pre- and post-intervention. The questions were a subset of the questions the participants answered during the 10-week program. Questions were derived directly from publicly available material on websites from the Centers for Disease Control (CDC), Arthritis Foundation, and National Institute on Aging (NIA).

### 2.3 Intervention

The gameplay and design of the Bingocize® app has been described in several previous studies, and so will not be repeated here in detail [18]. The data collected for the present manuscript were part of a larger investigation described elsewhere [18].

The intervention used the Bingocize® app twice a week for about an hour per session for 10 weeks. The tablets (either Samsung Galaxy Tab 4 or Digiland 16 GB tablets with a 10.1” screen) used to play the game were provided to the participants. App software was hosted remotely on a dedicated Linux server at WKU. Each participant was supplied their own tablet, and the game progressed while participants were gathered around a large table. Each activity (physical exercise or answering a health education question) was associated with a unique number and those numbers were used to populate the virtual bingo cards. The Bingocize® app was designed to include each participant of a session in an activity for each called number. To accomplish this, the app shows all participants a bingo card with the *same* numbers, but for each participant the numbers are located in different (random) arrangements. Thus, each participant finds each “called” number on their card to complete the associated activity. When a number is called, participants first complete the related activity (exercise or health education question) and then mark the corresponding number on their card by selecting the number using the tablet’s touchscreen.

For an exercise activity (experimental group only), participants followed on-screen and verbal instructions from a “coach” to complete the exercise. The exercise component was designed using the American College of Sports Medicine position stand for exercise and physical activity guidelines for older adults [23]. Participants completed 12 exercises equally divided between endurance/balance, strength, and flexibility. Progressive resistive exercise was employed with participants progressing from performing eight repetitions per exercise each session to fifteen repetitions per exercise each session by the end of the intervention. Participants were encouraged to maintain a moderate level of intensity when performing the exercises (based on Ratings of Perceived Exertion; RPE).

For health education questions (both groups), participants were asked to select the correct answer to a given multiple-choice or true/false question. In order to ensure that the participant was able to learn the correct answer, incorrect respondents were instructed to try again until the correct one was selected. The coach expounded upon the given question by adding additional information and engaging the participants in discussion.

The game continued until one participant won the game; on average, four games were played per session to make sure that a sufficient number/selection of activities (exercises and/or health education questions) was completed. All sessions were facilitated by a trained staff member (“coach”) of the senior centers where the interventions took place. The staff member’s version of the app was different, allowing for monitoring of attendance and adherence, and control of the spinning ‘virtual’ wheel to randomly select the next number for the game. Random sessions were also attended intermittently by members of the research team to ensure fidelity of the program.

### 2.4 Health Education Component

A set of 100 multiple-choice and true/false questions was the basis for the health education component. The experimental and control groups answered a total of 360 questions over the 20 sessions, which means each question was encountered and answered approximately 3.6 times. The questions covered two areas: fall risk reduction and osteoarthritis. There were 50 questions for each topic, 10 of which were true/false questions for the chronic disease management (osteoarthritis) topic. The topics were selected because they cover recognized areas of importance to older adults [23]. In addition to the questions the participants viewed on their app, for each question the coach’s app showed additional information which coaches could share with the participants.

Here is an example question from the fall risk reduction topic:

Question: What is the percentage of falls that happen every day in the home due to overlooked hazards?

Answer: 50–75% of falls happen in the home every day due to overlooked hazards.

(The given answer is the correct answer.Other answers used either lower or higher percentages.)

Here is an example multiple-choice question from the osteoarthritis area:

Question: What is a low-impact option for endurance exercise?

Answers: a. Running, b. Jumping jacks, c. **Swimming** d. Jumping rope.

(The correct answer is given here in bold.)

### 2.5 Analysis

A two (Group: Exercise + Health Education vs. Health Education-only) x two (Time: Pre- vs. Postintervention) analysis of variance, with significance set to *p<*.05, was used to detect any significant pre- to post-intervention changes in PAM-10 and health education knowledge test scores. Effect sizes are reported as partial eta squared values $\left(\eta_{p}^{2}\right)$. The Statistical Package for the Social Sciences (SPSS 24) was used for analysis.

The random assignment of participants resulted in two homogeneous groups completing the study and no significant differences in age, education, mental status, or body weight between the completing participants and the dropouts were found using chi-square analysis. The mean age of participants was 73.43 years, 86% were female and 76% were Caucasians and approximately 80% had a high school diploma or less as their highest form of education. The mean Telephone Mini Mental Status Examination (T-MMSE) score was 19.67 ± 1.35.

## 3. Results

The analysis of the PAM-10 questionnaire data obtained evidence the 10-week program led to an increase in the PAM-10 scores in both groups F(1,82)=8.29, p=.005, $\eta_{p}^{2}$ = .09, however, there were no between-group differences in PAM-10 scores F(1,82)= .002, p=.962, $\eta_{p}^{2}$ = .000. Similarly, both groups improved their knowledge related to falls reduction and osteoarthritis F (1,83)=275.56, p=.000, $\eta_{p}^{2}$ *=* .77 and there were no between-group differences F(1,83)=.416, p=.521, $\eta_{p}^{2}$ *=* .005. Table 2 shows means and standard errors for the outcome measures, broken down by group and time.

## 4. Discussion

We sought to determine if a multimodal version of Bingocize® that combines exercise and health education shows greater increases in health activation than a unimodal approach that uses only health education. The present investigation provides important evidence that engaging in a multimodal *or* unimodal game-centered health promoting activity can lead to higher levels of health activation in community-dwelling older adults. This is important because other studies have reported that increases in health activation predict decreases in hospitalization and increases in medication adherence [6].

In our study, both groups’ adherence (attendance) was high and very similar (94% in the Exercise Group and 93% in the Health Education-only group). This level of adherence is promising because it is greater than what is typically found in other health intervention studies [23], A major barrier to improving older adults’ health and well-being is motivating them to adhere to health-promoting programs. Older adults’ selection of leisure time activities is often motivated by specific motivations or needs such as cognitive, connectedness, physical motivation, accomplishment, individuality, affect, and escapism [24]. Bingocize® touches on a variety of these needs and thus older adults may be more willing to participate.

Seventy-nine percent (79%) of the participants had a PAM-10 score at levels 3 or 4 for the pretest. That is in line with data from a large-scale study where roughly 4 out of 5 patients scored in the top two levels [25], Approximately 92% of the participants scored at levels 3 and 4 during the post-test. Both groups significantly improved their knowledge of osteoarthritis and falls risk reduction which likely contributed to the improvements in health activation. These results are consistent with others who found the Chronic Disease Self-Management Program was capable of increasing health activation, as well as physical activity, by education alone [26].

In this study, it was surprising the addition of exercise to the health education component did not result in significantly higher PAM-10 levels compared to health education alone. However, when Blocker [27] compared an education-only group to a combined education and exercise group, both groups increased daily physical activity (footsteps), but there were no between group differences. He suggested the motivational climate and interactive style of the education motivated the rural adults to increase physical activity. In this investigation, the *main* focus of the health topics (osteoarthritis and falls prevention) in both conditions was not increasing physical activity. Yet, even more surprising was the fact both groups improved chair stand performance (a test of lower body strength) at the end of the intervention (Shake et al., 2018). We speculate because Bingocize® is game-centered and interactive, the older adults were motivated to increase their physical activity levels outside of the intervention.

The groups’ high adherence in our study is important considering participants were recruited from rural Tennessee and Kentucky where low socioeconomic status and lower education are common. In fact, 80% of our participants had a high school diploma or less as their highest form of education. Of the 10 participants that moved to a higher PAM-10 level after the intervention, nine graduated from high school and one earned an Associate’s degree. These results suggest the content and difficulty of the health information presented during Bingocize® was appropriate and sufficient to lead to increased health activation of the less educated participants

Additional investigation is needed to determine the exact mechanisms by which Bingocize® encourages high adherence, but we speculate because the program utilizes components of Social Cognitive Theory (modelling and self-efficacy) and Self-Determination Theory (SDT), participants are intrinsically and extrinsically motivated to join and continue the program [28]. This is consistent with others who found an increase in physical activity minutes after a web-based program that combined SDT and other traditional health behaviour theories [29]. Also, although we did not formally assess participants’ perceptions of the program, many expressed how much they enjoyed the social aspect of Bingocize®. In previous studies, older adults were found to place a high level of emphasis on the emotional and social satisfaction from routine physical activity. Those who consistently participate in exercise enjoy and expect a social connection with a group, novel types of exercise, and to be a part of a larger community [30, 31].

Of course, a limitation of the present study is that we do not have data about whether participants’ health activation stayed higher after the intervention ended, or whether they translated these improved psychological components into actual behaviour changes. However, there is evidence suggesting health activation can be accompanied by changes in self-management behaviours including exercise [26]. Future research will need to be conducted to assess those connections and compare them to other studies using the PAM.

## 5. Conclusions

Bingocize® engages older adults in a game-centered health promotion program within a familiar and fun context, encouraging adherence and, in turn, increasing their PAM-10 scores. Bingocize® offers a way to overcome traditional healthcare barriers and encourages older adults to engage in exercise and health education. Participants’ health activation was improved after the 10-week intervention, however, additional research is needed to examine whether changes in health activation results in long-term health behaviour changes. Multiple versions of Bingocize® can be successfully used to improve older adult’s knowledge, skills, and confidence to manage one’s healthcare. The social interactions during the program might attract and retain older adults who would not participate in traditional health promotion programs.

## Figures and Tables

**Table 1:** Baseline pre-intervention demographics of cluster randomized participants.

	Experimental (n=60)	Control (n=45)
Sex		
Male	9	6
Female	51	39
Age (years)	73.59 (7.87)	73.22 (7.79)
Race & Ethnicity		
Caucasian	43	37
African-American	9	4
American Indian	2	0
Hispanic/Latino	2	0
Other	1	0
Not Reported	3	4
Education (Highest)		
Less than high school	3	3
High school	43	28
Associate’s degree	4	8
Bachelor’s degree	3	0
Graduate degree	3	1
Not Reported	4	5
Anthropometries & Mental State		
Height (cm)	159.28(10.37)	155.75 (12.66)
Mass (kg; Pre-intervention)	80.26 (20.58)	84.87 (19.47)
BMI (Pre-intervention)	31.81(8.57)	35.40 (9.46)
T-MMSE (max=21)	19.81 (1.41)	19.50(1.19)
Self-Reported Health Conditions		
Diabetes*	14 [23%]	19 [42%]
High Blood Pressure	39 [65%]	33 [73%]
High Cholesterol	33 [55%]	26 [58%]

Numbers in parentheses () are standard deviations; in brackets [] are percentages; *BMI*, Body Mass Index; *T-MMSE*, Mini-Mental State Examination;

* indicates significant difference at baseline, *p* < .05.

**Table 2:** Means (and standard errors) for outcome measures among completed participants.

Outcome Measures	Experimental (n=47)			Control (n= 37)		
	Pre	Post	+/−	Pre	Post	+/−
PAM-10 scores^#^	64.27 (1.92)	68.75 (2.05)	+4.48	63.09 (2.19)	67.72 (2.33)	+4.63
Health Knowledge Test*	18.72 (.54)	23.62 (.53)	+4.90	17.95 (.52)	23.32 (.52)	+5.37

# Notes: Patient Activation Measure; scored on a 0–100 scale.

* Mean number of correct answers out of 30 questions.
